# Empiric Antibiotic Therapy in Suspected Sepsis: Impact of Gentamicin-Based Regimens on Incident Renal Failure and Mortality

**DOI:** 10.1093/ofid/ofaf319

**Published:** 2025-06-04

**Authors:** Magrit Jarlsdatter Hovind, Jan Erik Berdal, Olav Dalgard, Magnus Nakrem Lyngbakken

**Affiliations:** Department of Infectious Diseases, Akershus University Hospital, Lørenskog, Norway; Institute for Clinical Medicine, University of Oslo, Oslo, Norway; Department of Infectious Diseases, Akershus University Hospital, Lørenskog, Norway; Institute for Clinical Medicine, University of Oslo, Oslo, Norway; Department of Infectious Diseases, Akershus University Hospital, Lørenskog, Norway; Institute for Clinical Medicine, University of Oslo, Oslo, Norway; Department of Infectious Diseases, Akershus University Hospital, Lørenskog, Norway; Institute for Clinical Medicine, University of Oslo, Oslo, Norway

**Keywords:** antibiotics, antimicrobial stewardship, mortality, nephrotoxicity, sepsis

## Abstract

**Background:**

The efficacy and safety of administering a narrow-spectrum β-lactam and gentamicin as empirical therapy for community-acquired sepsis has been questioned. We compared the efficacy and safety of this combination with that of broad-spectrum β-lactams (cefotaxime, piperacillin-tazobactam, or meropenem) in patients with suspected sepsis.

**Methods:**

In this retrospective study, we included patients initiated on narrow-spectrum β-lactam/gentamicin or broad-spectrum β-lactams for suspected sepsis between January 2017 and December 2022. Patients without baseline creatinine and at least 1 subsequent creatinine measurement were excluded. We compared the impact of antibiotic regimens using a 5-level ordinal outcome scale ranging from no acute kidney injury (AKI) to all-cause death during 30-day follow-up.

**Results:**

Among 1917 patients, 33.1% received narrow-spectrum β-lactam/gentamicin, and 66.9% received broad-spectrum β-lactams. Patients initiated on broad-spectrum β-lactams had more comorbidities, had lower estimated glomerular filtration rate on admission, and more frequently required treatment with noradrenaline, respiratory support, and admission to the intensive care and medical intermediate care units. Therapy with broad-spectrum β-lactams was associated with a higher posttreatment stage of AKI or death (adjusted odds ratio, 1.61 [95% confidence interval, 1.27–2.04]). We found no significant association between cumulative dose of gentamicin and peak creatinine value. For patients treated with gentamicin experiencing AKI, creatinine normalized during 30-day follow-up.

**Conclusions:**

In patients with suspected sepsis, empirical treatment with narrow-spectrum β-lactam/gentamicin was not associated with an increased risk of AKI or death. If local antimicrobial resistance patterns permit, narrow-spectrum β-lactam/gentamicin may reduce broad-spectrum β-lactam usage, addressing a key element of antibiotic stewardship.

## Teaser

Empirical narrow-spectrum β-lactam/gentamicin was not associated with an increased risk of acute kidney injury (AKI) or death. In patients experiencing AKI, creatinine normalized within 30 days. If local antimicrobial resistance patterns permit, narrow-spectrum β-lactam/gentamicin may reduce broad-spectrum β-lactam usage.

The importance of early recognition and rapid initiation of empiric antibiotic therapy in patients with sepsis is widely recognized [1] and has been shown to improve outcomes [2]. The choice of empirical antibacterial therapy remains debated, as an ideal regimen should both ensure broad coverage for early appropriate treatment and aim to limit the use of broad-spectrum β-lactams that unproportionally contribute to the emergence of antimicrobial resistance (AMR). Recommended empirical antibiotics in suspected sepsis differ between countries, reflecting local resistance patterns [1] and different antibiotic policies. Norway is considered to be a low-AMR-setting country. In 2023, national surveillance reported that resistance patterns from blood culture isolates of Enterobacterales such as *Escherichia coli* and *Klebsiella* species commonly associated with sepsis showed 4%–5% resistance for gentamicin and 8%–10% for ciprofloxacin, and 5%–6% were reported as extended-spectrum β-lactamase (ESBL) producing. There was a slight increase to 1.8% in the proportion of methicillin-resistant *Staphylococcus aureus* (MRSA) in blood cultures, from 0.8% in 2021 and 1% in 2022, and only 0.3% of *Streptococcus pneumoniae* from blood and spinal fluid cultures were penicillin resistant [3]. In the national Norwegian guidelines, a narrow-spectrum β-lactam (benzylpenicillin, ampicillin) combined with gentamicin is recommended as first-choice empiric therapy in patients with community-acquired sepsis. The penicillins provide coverage for streptococcal species and gentamicin coverage for Enterobacterales. Recommended alternative broad-spectrum regimens include third-generation cephalosporins, extended-spectrum β-lactams with β-lactamase inhibitors, and carbapenems. The guidelines suggest considering the latter alternative regimens if there are contraindications to the use of gentamicin (ie, severely impaired renal function, renal transplant, concomitant use of nephrotoxic medications) or if high risk of antimicrobial resistance such as known colonization/infection with resistant microbes in the past 12 months, recent treatment with broad-spectrum antibiotics, or travel history to countries with a high prevalence of antimicrobial resistance [4]. Serum concentration measurements of gentamicin are recommended before the second dose in certain cases, that is, estimated glomerular filtration rate (eGFR) <30 mL/minute/1.73 m^2^, acute change in renal function, and critical illness, among others. Otherwise, serum concentration measurements are recommended after the third dose [5]. As empiric regimens are typically only prescribed for the first 24–48 hours of admission followed by de-escalation based on suspected focus of infection and microbiological results, most patients do not have concentration measurements performed.

Aminoglycosides have several beneficial pharmacodynamic effects for the treatment of sepsis [6, 7]. They have minimal penetration into the gut lumen [8], thereby reducing antibiotic selective pressure on the gut microbiota and possibly reducing risk for *Clostridoides difficile* infection and development of AMR. A synergistic effect is achieved when combined with penicillin, which has minimal impact on the gram-negative anaerobes and Enterobacterales of the gut flora [3]. Few studies have compared narrow-spectrum β-lactam and gentamicin with more broad-spectrum alternatives, and numerous studies have demonstrated low risk of nephrotoxicity after introduction of gentamicin with once-daily dosing [9] and short-course treatment [10–13], as recommended in the Norwegian guidelines [4].

To limit the emergence of AMR, the use of broad-spectrum β-lactams must be reduced. Further evidence on whether narrow-spectrum β-lactam/gentamicin therapy is safe and effective is therefore needed. Analyzing a large cohort of patients with suspected sepsis, we compared the outcomes of empiric narrow-spectrum β-lactam/gentamicin and broad-spectrum β-lactams.

## METHODS

### Study Design and Patient Population

**Figure 1.**
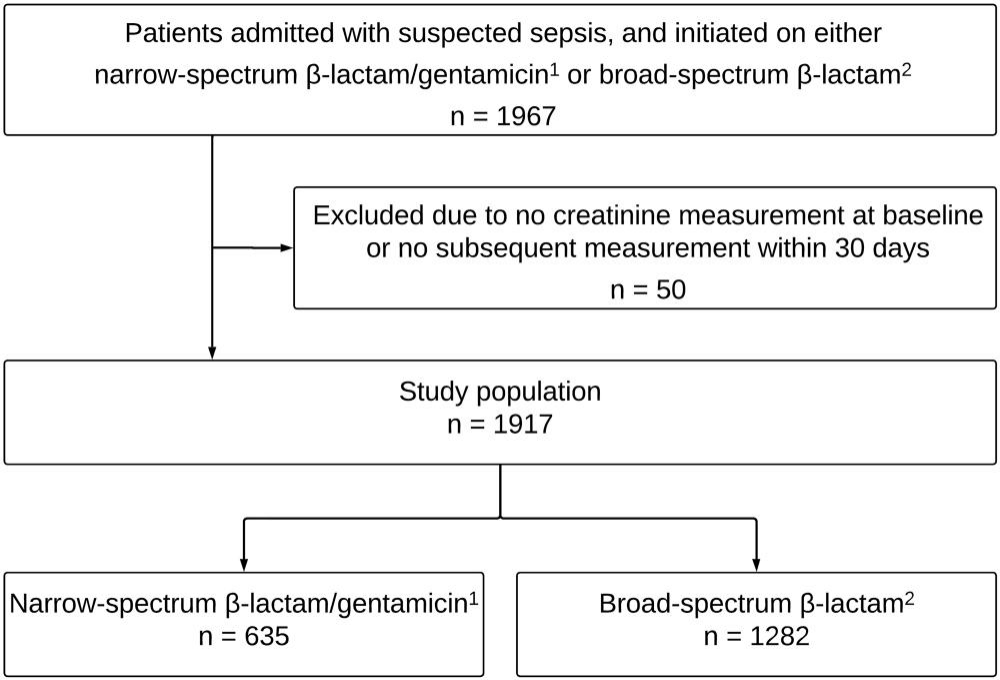
Flow diagram of study inclusion (study period from 1 January 2017 to 31 December 2022). ^1^Narrow-spectrum β-lactams include benzylpenicillin and ampicillin. ^2^Broad-spectrum β-lactams include cefotaxime, piperacillin-tazobactam, and meropenem.

This was a retrospective study conducted at Akershus University Hospital, Lørenskog, the largest emergency care hospital in Norway, serving a population of approximately 600 000 and managing 46 000 emergency admissions per year. We included adult patients ≥18 years of age admitted to the emergency department (ED) from the community with suspected sepsis in the study period between 1 January 2017 and 31 December 2022. Due to lack of data on recent hospitalizations, some healthcare-associated infections may have been included in the study. Patients with suspected sepsis were identified retrospectively by the data warehouse through the electronical medical record based on the registered need of immediate attendance upon arrival to the ED by the medical emergency team or sepsis response team. These teams of experienced physicians and emergency nurses have been established to ensure timely early recognition and treatment of sepsis and are initiated by the paramedics or the receiving physicians in the ED according to specific criteria indicative of sepsis (such as quick Sequential Organ Failure Assessment score [14] of ≥2) or critical disease. We included patients assessed by the medical emergency team or sepsis response team if they were initiated on empiric sepsis treatment with a narrow-spectrum β-lactam (benzylpenicillin or ampicillin) and gentamicin or 1 of the following broad-spectrum alternatives: cefotaxime, piperacillin-tazobactam, or meropenem (Figure 1). Patients receiving at least 1 dose of these antibacterial therapies were included. Inclusion in the study was based on suspicion of sepsis and initiation of empiric sepsis therapy, regardless of whether the patients ultimately received a diagnosis of infection or sepsis at discharge. To enable assessment of acute kidney injury (AKI), we excluded patients without a baseline creatinine and at least 1 subsequent creatinine measurement within 30 days after admission.

### Data Collection

All data, originally collected for clinical purposes, were extracted automatically from the electronical medical record between June 2023 and June 2024 by the local data warehouse. Retrieved data include demographics, current medication, vital signs at admission, laboratory results including all creatinine measurements performed up to 30 days after admission to the ED, blood cultures, antibiotic therapy, and date of death. Blood cultures were collected as indicated clinically, typically from all patients assessed by the medical emergency team or sepsis response team. We retrieved data on all positive blood cultures, but positive blood cultures with bacteria typically considered contaminants, such as coagulase-negative staphylococci, were considered negative. For antibiotic therapy, we extracted antibacterial agents and total dose prescribed during the hospital stay. Body weight data were not systematically registered in the medical records, but during the study period the Norwegian guidelines recommended gentamicin dosed 5–7 mg/kg once daily (current recommendation since 2022 is 6–7 mg/kg) and gentamicin was assumed to be prescribed according to weight. Data on treatment with vasopressor (noradrenaline), renal replacement therapy, respiratory support (high-flow oxygen, noninvasive ventilation, or ventilator), and admission to intensive care unit (ICU) or medical intermediate care unit were extracted based on procedure codes registered for the current admission. The hospital medical intermediate care unit receives patients requiring a higher level of monitoring and intensive treatment than is possible in the regular wards, frequently in need of organ support such as vasopressor, noninvasive ventilation, or short-term ventilator treatment. Data on specific comorbidities were obtained from extracting all discharge diagnoses (*International Classification of Diseases, Tenth Revision*) from all historical hospital admissions and outpatient visits. Based on these, a Charlson Comorbidity Index (CCI) score [15] was calculated for each patient, and the discharge diagnoses of the current admission were used to determine whether the patient was diagnosed with an infection at discharge, and if so, the focus of infection. If missing, National Early Warning Score 2 (NEWS2) [16] values were imputed using predictive mean matching.

### Outcomes

The clinical outcomes of interest in this study were all-cause mortality and kidney injury, occurring after exposure to the antibacterial agents. We calculated the difference in creatinine from baseline, defined as the first creatinine measurement within 3 days of admission, to the highest creatinine measured within 30 days after admission to the ED. Although time to AKI after treatment initiation with aminoglycosides is typically 5–10 days, we chose a timeframe of 30 days to include all potential gentamicin-associated AKI. We used a 5-level ordinal scale ranging from no AKI to all-cause death as the primary study outcome, as previously used in the Antibiotic Choice on Renal Outcomes (ACORN) study for assessment of nephrotoxicity in the comparison of cefepime and piperacillin-tazobactam [17]. The stages of AKI are based on the Kidney Disease: Improving Global Outcomes criteria for creatinine level (KDIGO) criteria [18], which classifies AKI into stages 1–3. The stages of the 5-level ordinal scale used in this study are as follows: stage 0, survival and no AKI; stage 1, survival and KDIGO stage 1 (creatinine increase by 1.5–1.9 times baseline or ≥26.5 µmol/L); stage 2, survival and KDIGO stage 2 (creatinine increase by 2.0–2.9 times baseline); stage 3, survival and KDIGO stage 3 (creatinine increase by ≥3.0 baseline or ≥353.7 µmol/L, or receipt of acute kidney replacement therapy); and stage 4, death. All-cause mortality was included in the ordinal outcome scale to account for competing risk of death. In the ACORN study, the primary outcome based on this novel ordinal scale was evaluated on day 14 after randomization, whereas we prolonged the observational time as described above. We further examined the dose-response between total dose of antibacterial agent prescribed and the peak creatinine level measured up to 30 days after admission to the ED. Last, to assess persistent kidney injury, we evaluated the 30-day creatinine trajectories for patients who did and did not experience at least an increase in creatinine ≥1.5 times baseline.

### Statistical Analysis

Demographics and baseline characteristics are presented as absolute numbers (proportion) or median (interquartile range [IQR]), and the comparisons of the empiric antibiotic regimens were performed using the Mann-Whitney *U* test or χ^2^ test as appropriate. The numbers and proportions of patients experiencing each stage of the primary outcome are presented descriptively according to antibiotic regimen. We used ordinal logistic regression to assess the association of antibiotic regimen on mortality and AKI, and adjusted the analyses for sex, age, CCI score, baseline kidney function (eGFR), time to first dose of antibiotics, and markers of disease severity including NEWS2 score at admission, treatment with noradrenaline, respiratory support, and admission to ICU or medical intermediate care unit. As a sensitivity analysis, we evaluated the clinical outcome by combining stage 1–3 of the ordinal scale (survival with all stages of AKI), and separating stage 4 into death without AKI and death with any stage of AKI. We performed interaction analysis to assess effect modification by kidney function (above/below normal eGFR, defined as ≥60 mL/minute/1.73 m^2^). The patients in this cohort were included based on clinical suspicion of sepsis, rather than using a precise sepsis definition. As an overestimation of sepsis could potentially affect the results, we performed a sensitivity analysis excluding patients without a diagnosis of infection at discharge. To further account for confounding by indication and make the comparison between the 2 treatment groups more reliable, we performed a sensitivity analysis using inverse probability of treatment weighting (IPTW) accounting for the adjustment covariates. For the dose-response assessment, we adjusted for baseline creatinine and all the same adjustment variables as in the main analysis. To account for nonlinear associations, we modeled dose of antibiotics with restricted cubic splines with knots placed at the 10th, 50th, and 90th sample percentiles. The creatinine trajectories up to 30 days after admission to the ED are presented according to empiric antibiotic regimens separately, and patients were grouped according to whether they experienced an increase in creatinine ≥1.5 times baseline. The trajectories of creatinine were modeled using a generalized linear mixed model with subject-specific random intercept and slope, and were additionally adjusted for renal replacement therapy, as this covariate was not included in the definition of AKI used in this model. There were no missing values for any adjustment variables in the analyses except for NEWS2 score, which was imputed with predictive mean matching if missing. The statistical analyses were performed using Stata version 17 software (StataCorp LP, College Station, Texas, USA). For all analyses, a *P* value of <.05 was considered significant.

### Ethics

The study was approved by the Data Protection Officer at Akershus University Hospital and the Norwegian Directorate of Health, with a waiver of informed patient consent (reference number 22/47245-9).

## RESULTS

### Baseline Characteristics, Antibiotic Therapy, and Source of Infection

After exclusion of 50 patients due to missing creatinine measurements, the final cohort included 1917 patients admitted to the ED with suspected sepsis and initiated on 1 of the antibiotic regimens between 1 January 2017 and 31 December 2022 (Figure 1). The median age was 75 (IQR, 65–82) years and 60.6% (n = 1161) were male.

Combination therapy with narrow-spectrum β-lactam/gentamicin was administered to 33.1% of patients (n = 635/1917), and patients initiated on this regimen received a median of 2 doses of gentamicin (Supplementary Table 1). Among the patients who received broad-spectrum β-lactams (66.9% [n = 1282/1917]), 82.9% (n = 1063/1282) received cefotaxime, 15.5% (n = 199/1282) piperacillin-tazobactam, and 1.6% (n = 20/1282) meropenem. In comparison with patients initiated on narrow-spectrum β-lactam/gentamicin, those who received broad-spectrum β-lactams were more comorbid with a higher CCI score, had a lower eGFR at admission, and more frequently required treatment with noradrenaline, respiratory support, and admission to the ICU or medical intermediate care unit (Table 1).

**Table 1.** Baseline Characteristics, Infection, and Disease Severity According to Empirical Antibiotic Therapy Regimen

Characteristic	Narrow-Spectrum β-Lactam/Gentamicin (n = 635)	Broad-Spectrum β-Lactams (n = 1282)	*P* Value
Male sex	381 (60.0)	780 (60.8)	.72
Age, y	75.0 (67.0–82.0)	75.0 (64.0–82.0)	.34
Systolic blood pressure, mm Hg	125 (105–143)	123 (98–143)	.050
Diastolic blood pressure, mm Hg	67 (58–81)	67 (56–81)	.25
Respiratory rate, breaths per minute	28 (24–32)	28 (23–35)	.90
Heart rate, beats per minute	106 (90–122)	104 (86–120)	.046
Temperature, °C	38.0 (37.1–38.8)	37.5 (36.5–38.4)	<.001
Oxygen saturation, %	93 (88–96)	93 (87–96)	.59
Supplemental oxygen	343 (54.0)	732 (57.1)	.20
Comorbidities			
Charlson Comorbidity Index score	2 (1–3)	2 (1–5)	<.001
Myocardial infarction	91 (14.3)	225 (17.6)	.074
Congestive heart failure	101 (15.9)	313 (24.4)	<.001
Cerebrovascular disease	124 (19.5)	258 (20.1)	.76
Dementia	49 (7.7)	86 (6.7)	.42
Chronic pulmonary disease	196 (30.9)	365 (28.5)	.28
DM without complications	105 (16.5)	263 (20.5)	.037
DM with complications	20 (3.1)	112 (8.7)	<.001
Chronic kidney disease	23 (3.6)	215 (16.8)	<.001
Any malignancy	147 (23.1)	362 (28.2)	.018
Metastatic solid tumor	54 (8.5)	108 (8.4)	.95
Biochemistry			
C-reactive protein, mg/L	70 (27–142)	70 (22–160)	.64
Ferritin, μg/L	336 (135–587)	384 (164–1039)	.19
eGFR, mL/min/1.73 m^2^	77 (54–90)	48 (29–78)	<.001
Creatinine, μmol/L	80 (64–101)	116 (78–179)	<.001
Creatinine, mg/dL	0.9 (0.7–1.1)	1.3 (0.9–2.0)	<.001
White blood count, × 10^9^ cells/L	12.3 (8.9–16.5)	12.2 (8.4–16.8)	.53
Lactate, mmol/L	1.4 (1.0–2.3)	1.8 (1.1–3.2)	<.001
Infection			
Infectious disease cause of admission	514 (80.9)	944 (73.6)	<.001
Respiratory tract	323 (50.9)	615 (48.0)	.23
Urinary tract	126 (19.8)	189 (14.7)	.005
Bacteremia^a^	92 (14.5)	222 (17.3)	.12
Disease severity			
NEWS2	8 (6–10)	8 (6–9)	.094
Admission to ICU or medical intermediate care unit	147 (23.1)	522 (40.7)	<.001
Noradrenaline	72 (11.3)	341 (26.6)	<.001
Respiratory support	130 (20.5)	357 (27.8)	<.001

Data are presented as median (interquartile range) for continuous measures or No. (%) for categorical measures.

Abbreviations: DM, diabetes mellitus; eGFR, estimated glomerular filtration rate; ICU, intensive care unit; NEWS2, National Early Warning Score 2.

^a^Positive blood cultures with bacteria considered to represent contamination, such as coagulase-negative staphylococci, are excluded.

At discharge, 76.1% (n = 1458) were diagnosed with an infection, with the respiratory tract and urinary tract as the most prevalent infection foci; 16.4% (n = 314) had a positive blood culture, with similar rates of bacteremia in the 2 treatment groups (Table 1, Supplementary Table 2). A final diagnosis of infection was less frequent in patients initiated on broad-spectrum β-lactams than patients initiated on narrow-spectrum β-lactam/gentamicin (73.6% vs 80.9%, respectively; *P* value for comparison <.001).

### Outcomes

**Figure 2.**
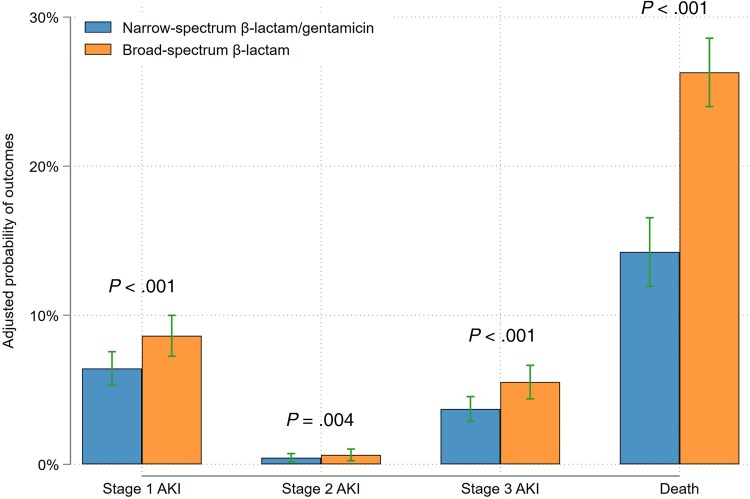
Adjusted probabilities of clinical outcomes according to antibiotic regimen. Probabilities derived from ordinal logistic regression. Adjusted for age, sex, Charlson Comorbidity Index score, kidney function (estimated glomerular filtration rate), time to first dose of antibiotics, and markers of disease severity including National Early Warning Score 2, noradrenaline, respiratory support, and admission to intensive care unit or medical intermediate care unit. Abbreviation: AKI, acute kidney injury.

**Figure 3.**
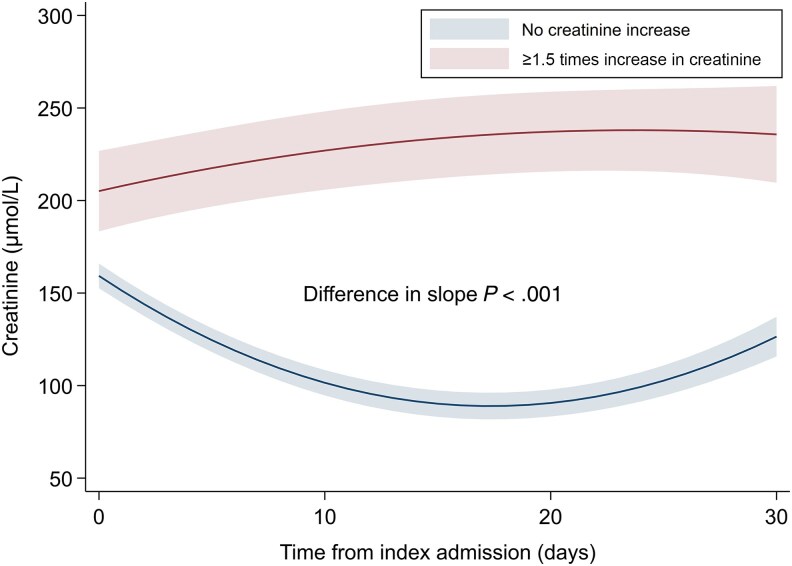
Changes in creatinine values during 30 days after admission for patients receiving broad-spectrum β-lactams. Patients with no creatinine increase compared to patients with ≥1.5 times increase in creatinine within 30 days after admission according to empiric antibiotic regimen. Adjusted for age, sex, Charlson Comorbidity Index score, renal replacement therapy, time to first dose of antibiotics, and markers of disease severity including National Early Warning Score 2, noradrenaline, respiratory support, and admission to intensive care unit or medical intermediate care unit.

**Figure 4.**
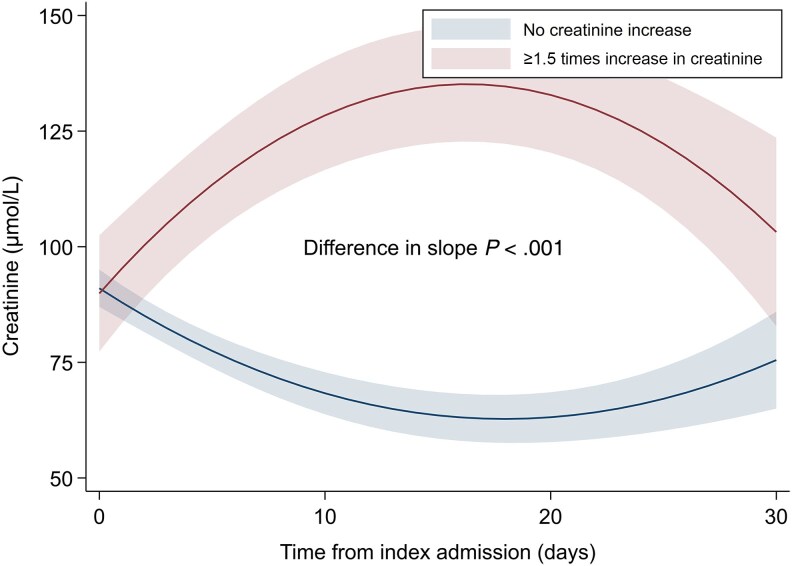
Changes in creatinine values during 30 days after admission for patients receiving narrow-spectrum β-lactam/gentamicin. Patients with no creatinine increase compared to patients with ≥1.5 times increase in creatinine within 30 days after admission according to empiric antibiotic regimen. Adjusted for age, sex, Charlson Comorbidity Index score, renal replacement therapy, time to first dose of antibiotics, and markers of disease severity including National Early Warning Score 2, noradrenaline, respiratory support, and admission to intensive care unit or medical intermediate care unit.

Overall, the unadjusted all-cause 30-day mortality rate was 23.1% (n = 442), with a significantly higher mortality rate in patients initiated on broad-spectrum β-lactams (26.9% [n = 345/1282] vs 15.3% [n = 97/635]; *P* value for comparison <.001). The unadjusted numbers and proportions for all stages of the primary outcome according to antibiotic regimen are outlined in Table 2. The proportion of patients who experienced any stage of AKI or death was 41.8% (n = 536/1282) of patients prescribed broad-spectrum β-lactams and 24.4% (n = 155/635) of patients initiated on narrow-spectrum β-lactam/gentamicin. In adjusted analyses, initiation of broad-spectrum β-lactams was associated with a higher level of AKI or death (odds ratio [OR], 1.61 [95% confidence interval {CI}, 1.27–2.04]; Figure 2). Similar results were observed when evaluating death with and without AKI separately (Supplementary Figure 1). There was no effect modification of baseline kidney function (eGFR above or below 60 mL/minute/1.73 m^2^) on the association between antibiotic regimen and the clinical outcome (*P* value for interaction >.05). Initiation of broad-spectrum β-lactams was also associated with a higher level of AKI or death when including only patients with a final diagnosis of infection (adjusted OR [aOR], 1.58 [95% CI, 1.21–2.07]; Supplementary Table 3) and when using IPTW (aOR, 1.81 [95% CI, 1.37–2.39]). There was no significant association between cumulative dose of broad-spectrum β-lactams or gentamicin and peak creatinine value measured within 30 days (Supplementary Figure 2). For patients who experienced at least a 1.5 times increase in creatinine during 30-day follow-up, mean creatinine levels remained slightly elevated for patients receiving broad-spectrum β-lactams (Figure 3), but normalized during follow-up for patients receiving gentamicin (Figure 4).

**Table 2.** Associations Between Antibiotic Regimen and Primary Outcome

Outcome	Narrow-Spectrum β-Lactam/Gentamicin (n = 635)	Broad-Spectrum β-Lactams (n = 1282)	Adjusted OR^a^ (95% CI)
Primary outcome^b^, No. (%)			1.61 (1.27–2.04)
Stage 0	480 (75.6)	746 (58.2)	…
Stage 1	44 (6.9)	100 (7.8)	…
Stage 2	6 (0.9)	4 (0.3)	…
Stage 3	8 (1.3)	87 (6.8)	…
Stage 4	97 (15.3)	345 (26.9)	…

Abbreviations: CI, confidence interval; OR, odds ratio.

^a^Ordinal logistic regression. Adjusted for age, sex, Charlson Comorbidity Index score, kidney function (estimated glomerular filtration rate), time to first dose of antibiotics, and markers of disease severity including National Early Warning Score 2, noradrenaline, respiratory support, and admission to intensive care unit or medical intermediate care unit.

^b^Five-level ordinal scale: stage 0, survival and no creatinine increase; stage 1, creatinine increase by 1.5–1.9 times baseline or ≥26.5 µmol/L; stage 2, creatinine increase by 2.0–2.9 times baseline; stage 3, creatinine increase by ≥3.0 baseline or ≥353.7 µmol/L, or receipt of acute kidney replacement therapy; stage 4, death.

## DISCUSSION

In this large observational single-center study, we compared narrow-spectrum β-lactam/gentamicin and broad-spectrum β-lactams as empiric therapy for patients admitted to the ED with suspected sepsis. Our main finding is that empiric treatment with narrow-spectrum β-lactam/gentamicin was not associated with a higher stage of AKI or death compared with broad-spectrum β-lactams.

There is conflicting evidence regarding aminoglycoside combination therapy as empirical therapy for sepsis and bloodstream infections [10–13, 19–23]. Previous studies evaluating aminoglycoside combination therapy have used different and primarily broad-spectrum β-lactams, which provide adequate gram-negative coverage [19, 22]. Evidence on whether narrow-spectrum β-lactam lacking gram-negative coverage in combination with gentamicin, as recommended in the Norwegian guidelines [4], is comparable to monotherapy with broad-spectrum β-lactams is very limited. The majority of conducted studies have dosing regimens of gentamicin that are no longer in use, including twice-daily dosing and long-term therapy [19], as opposed to current practice of once-daily dosing with shorter treatment duration [4]. Although evidence suggests that we do not achieve a survival benefit by adding an aminoglycoside to broad-spectrum β-lactams [19, 22], combining gentamicin with a narrow-spectrum β-lactam could be beneficial by reducing the use of broad-spectrum β-lactams. Implementation of restrictive antibiotic guidelines recommending gentamicin in combination with narrow-spectrum rather than broad-spectrum β-lactams has been shown to be associated with reduction in AMR and *C difficile* infections without worsening clinical outcomes [23]. However, the paucity of evidence and the risk of gentamicin-induced nephrotoxicity [19, 21] have limited the use of this empirical regimen.

Here, we address this knowledge gap by evaluating both mortality and nephrotoxicity in a large cohort of unselected patients with suspected sepsis, reflecting a real-world clinical setting. Being treated with broad-spectrum β-lactam was associated with a higher posttreatment stage of AKI and death than being treated with narrow-spectrum β-lactam/gentamicin. The association remained consistent after applying IPTW. This result was primarily driven by increased mortality, while the risk of kidney injury was similar in the 2 treatment groups. Randomized data directly comparing narrow-spectrum β-lactam/gentamicin and broad-spectrum β-lactams in terms of nephrotoxicity are lacking, but several studies have evaluated the risk of kidney injury associated with aminoglycoside therapy in patients with sepsis and serious infections [10–13, 21, 22]. Ong et al reported an increased risk of AKI associated with the addition of gentamicin to third-generation cephalosporins as empirical therapy in an ICU population with severe sepsis or septic shock [21]. In contrast, in a systematic review and meta-analysis, Heffernan et al found that β-lactams with once-daily aminoglycoside did not increase the risk of AKI compared to β-lactam monotherapy [22]. This finding is consistent with our results and with several other observational studies assessing short-course or single dose of gentamicin [10–12]. Total dose and treatment duration are risk factors for gentamicin-induced kidney injury [24, 25]. In our cohort, we found no association between the cumulative dose of gentamicin prescribed and peak creatinine on follow-up, likely due to short treatment duration with a median of 2 doses, equivalent to 2 days of treatment. This study included a 30-day follow-up period after admission and exposure to gentamicin. Although certain patients developed AKI, our results indicate that the risk of persistent kidney injury is low with once-daily dosing of gentamicin and short treatment duration, as previously reported [10, 11]. The fear of gentamicin-induced AKI is 1 of the main reasons for clinicians to choose an alternative regimen in the ED. In our study, empiric narrow-spectrum β-lactam/gentamicin did not pose a major risk of renal injury, provided selection of patients without known preexisting kidney disease or severely impaired baseline renal function, as well as considering other contraindications and precautions.

Despite a large cohort of nearly 2000 patients, we cannot exclude the possibility that the study may be underpowered to detect smaller effect size in AKI. However, our regression analysis and the complementary IPTW analysis, addressing confounding by indication, provide a robust estimate. The higher mortality of patients receiving broad-spectrum β-lactams as empiric regimen negatively influencing the outcome for this group may be explained by residual confounding. These patients had a lower baseline eGFR on admission and a higher likelihood of comorbidities, thus possibly representing a group with higher frailty. It is likely that the clinical judgment of the receiving physician in the ED led to selection of sicker patients with a higher mortality risk to the broad-spectrum β-lactam group, which is not entirely reflected by the extensive adjustments in the outcome regressions.

The study has some limitations. First, this is a single-center study conducted in a low-AMR setting. The generalizability of the results may therefore be limited by local resistance patterns. Second, with retrospective automatic extraction of data, we do not know the reason for missing creatinine values or the specific cause of AKI, but this affects both antibiotic groups randomly. Third, residual confounding cannot be excluded and may explain the increased mortality in patients initiated on broad-spectrum β-lactams, especially pertaining to the lack of reliable data for patient frailty. However, we emphasize the comparable outcomes of gentamicin-based regimen rather than increased mortality of broad-spectrum β-lactams, and further adjustment would most likely not change this result. Fourth, as we included patients based on clinical suspicion of sepsis rather than using a specific definition of sepsis, 24% of the patients in the cohort were not discharged with a diagnosis of infection. This reflects the common clinical situation where empiric antibiotic therapy in the ED is initiated based on suspicion and not certainty of infection. We accounted for this overestimation of sepsis through the sensitivity analysis including only patients with a diagnosis of infection at discharge, with unchanged results. Fifth, we have no data on ototoxicity, which is another important adverse effect associated with gentamicin. Although most likely underreported, none of the patients in the cohort had been given a discharge diagnosis of ototoxic hearing loss. Sixth, we do not have data on whether the patients in our cohort have had resistant bacteria detected previously, which may have influenced the treating physician's choice of empiric therapy. Seventh, the proportion of positive blood culture findings was similar between the 2 antibiotic groups, but we do not have data on susceptibility results and the appropriateness of antibiotic therapy, which could have influenced mortality. However, the prevalence of multidrug-resistant organisms (eg, MRSA and ESBL-producing pathogens) in Norway is low [3], and the yearly published local hospital resistance patterns (data not shown) are in line with national resistance patterns. Last, the lack of data on nephrotoxic agents, prescribed both prior to hospital admission and during hospitalization, is a limitation.

In conclusion, in this large cohort of patients with suspected community-acquired sepsis, empiric therapy with narrow-spectrum β-lactam/gentamicin was not associated with a higher stage of AKI or death compared to broad-spectrum β-lactams. The gentamicin-based regimen, as currently used in Norway with a selection of eligible patients, once-daily dosing, and short treatment duration, appears to be safe and effective. These results should be reassuring for clinicians choosing this empiric antibiotic regimen for patients with suspected sepsis in the ED, may support reduction in broad-spectrum β-lactam usage, and ultimately contribute to antibiotic stewardship.

## Supplementary Material

ofaf319_Supplementary_Data

## Supplementary Data

Supplementary materials are available at *Open Forum Infectious Diseases* online. Consisting of data provided by the authors to benefit the reader, the posted materials are not copyedited and are the sole responsibility of the authors, so questions or comments should be addressed to the corresponding author.

## Notes

***Author contributions.*** Concept and design: All authors. Data acquisition: Automatic extraction by the Unit of Data Analysis, Division of Research and Innovation, Akershus University Hospital. Analysis and interpretation: All authors. Drafting of the manuscript: M. J. H. Critical revision of the manuscript: All authors.

***Acknowledgments.*** The authors thank the Unit of Data Analysis, Division of Research and Innovation, Akershus University Hospital, particularly Tore Gundersen and Kristine Lippestad, for help with data extraction.

***Data availability.*** Data are not publicly available.

## References

[1] Evans L, Rhodes A, Alhazzani W, et al Surviving Sepsis campaign: international guidelines for management of sepsis and septic shock 2021. Crit Care Med 2021; 49:e1063–143. 34605781 10.1097/CCM.0000000000005337

[2] Seymour CW, Gesten F, Prescott HC, et al Time to treatment and mortality during mandated emergency care for sepsis. N Engl J Med 2017; 376:2235–44. 28528569 10.1056/NEJMoa1703058 PMC5538258

[3] NORM/NORM-VET 2023. Usage of antimicrobial agents and occurrence of antimicrobial resistance in Norway. Tromsø/Ås/Oslo. 2024.

[4] Norwegian Directorate of Health. National guidelines for use of antimicrobial agents in hospitals. Oslo, Norway: Norwegian Directorate of Health, **2013**.

[5] National Center for Antibiotic Use in Hospitals. Procedure aminoglycosides for adults. Bergen, Norway: National Center for Antibiotic Use in Hospitals, **2023**.

[6] Davis BD . Bactericidal synergism between β-lactams and aminoglycosides: mechanism and possible therapeutic implications. Rev Infect Dis 1982; 4:237–45. 7051225 10.1093/clinids/4.2.237

[7] Craig WA . Optimizing aminoglycoside use. Crit Care Clin 2011; 27:107–21. 21144989 10.1016/j.ccc.2010.11.006

[8] Staley C, Vaughn BP, Graiziger CT, Sadowsky MJ, Khoruts A. Gut-sparing treatment of urinary tract infection in patients at high risk of *Clostridium difficile* infection. J Antimicrob Chemother 2016; 72:522–8. 27999027 10.1093/jac/dkw499 PMC6075516

[9] Nicolau DP, Freeman CD, Belliveau PP, Nightingale CH, Ross JW, Quintiliani R. Experience with a once-daily aminoglycoside program administered to 2,184 adult patients. Antimicrob Agents Chemother 1995; 39:650–5. 7793867 10.1128/AAC.39.3.650 PMC162599

[10] Carlsen S, Boel J, Jarløv JO, Gjørup I, Søborg C, Arpi M. The effect of short-course gentamicin therapy on kidney function in patients with bacteraemia—a retrospective cohort study. Eur J Clin Microbiol Infect Dis 2018; 37:2307–12. 30225746 10.1007/s10096-018-3376-6

[11] Cobussen M, Haeseker MB, Stoffers J, Wanrooij VHM, Savelkoul PHM, Stassen PM. Renal safety of a single dose of gentamicin in patients with sepsis in the emergency department. Clin Microbiol Infect 2020; 27:717–23. 10.1016/j.cmi.2020.06.030 32621972

[12] Liljedahl Prytz K, Prag M, Fredlund H, Magnuson A, Sundqvist M, Källman J. Antibiotic treatment with one single dose of gentamicin at admittance in addition to a β-lactam antibiotic in the treatment of community-acquired bloodstream infection with sepsis. PLoS One 2020; 15:e0236864. 32730359 10.1371/journal.pone.0236864 PMC7392313

[13] Picard W, Bazin F, Clouzeau B, et al Propensity-based study of aminoglycoside nephrotoxicity in patients with severe sepsis or septic shock. Antimicrob Agents Chemother 2014; 58:7468–74. 25288085 10.1128/AAC.03750-14 PMC4249539

[14] Singer M, Deutschman CS, Seymour CW, et al The Third International Consensus Definitions for Sepsis and Septic Shock (Sepsis-3). JAMA 2016; 315:801–10. 26903338 10.1001/jama.2016.0287 PMC4968574

[15] Charlson ME, Pompei P, Ales KL, MacKenzie CR. A new method of classifying prognostic comorbidity in longitudinal studies: development and validation. J Chronic Dis 1987; 40:373–83. 3558716 10.1016/0021-9681(87)90171-8

[16] Royal College of Physicians. National Early Warning Score (NEWS) 2. Standardizing the assessment of acute-illness severity in the NHS. London, UK: Royal College of Physicians, **2017**.

[17] Qian ET, Casey JD, Wright A, et al Cefepime vs piperacillin-tazobactam in adults hospitalized with acute infection: the ACORN randomized clinical trial. JAMA 2023; 330:1557–67. 37837651 10.1001/jama.2023.20583 PMC10576861

[18] Kellum JA . KDIGO clinical practice guideline for acute kidney injury. Kidney Int Suppl 2012; 2:1–138.

[19] Paul M, Lador A, Grozinsky-Glasberg S, Leibovici L. Beta lactam antibiotic monotherapy versus beta lactam-aminoglycoside antibiotic combination therapy for sepsis. Cochrane Database Syst Rev 2014; 2014:CD003344. 24395715 10.1002/14651858.CD003344.pub3 PMC6517128

[20] Deelen JWT, Rottier WC, Buiting AGM, et al Short-course aminoglycosides as adjunctive empirical therapy in patients with gram-negative bloodstream infection, a cohort study. Clin Microbiol Infect 2021; 27:269–75. 32387438 10.1016/j.cmi.2020.04.041

[21] Ong DSY, Frencken JF, Klein Klouwenberg PMC, et al Short-course adjunctive gentamicin as empirical therapy in patients with severe sepsis and septic shock: a prospective observational cohort study. Clin Infect Dis 2017; 64:1731–6. 28329088 10.1093/cid/cix186

[22] Heffernan AJ, Sime FB, Sun J, et al β-lactam antibiotic versus combined β-lactam antibiotics and single daily dosing regimens of aminoglycosides for treating serious infections: a meta-analysis. Int J Antimicrob Agents 2020; 55:105839. 31704215 10.1016/j.ijantimicag.2019.10.020

[23] Ritchie ND, Irvine SC, Helps A, Robb F, Jones BL, Seaton RA. Restrictive antibiotic stewardship associated with reduced hospital mortality in gram-negative infection. Qjm 2017; 110:155–61. 27521583 10.1093/qjmed/hcw134

[24] Paterson DL, Robson JM, Wagener MM. Risk factors for toxicity in elderly patients given aminoglycosides once daily. J Gen Intern Med 1998; 13:735–9. 9824518 10.1046/j.1525-1497.1998.00224.x PMC1497032

[25] Bertino JS Jr, Booker LA, Franck PA, Jenkins PL, Franck KR, Nafziger AN. Incidence of and significant risk factors for aminoglycoside-associated nephrotoxicity in patients dosed by using individualized pharmacokinetic monitoring. J Infect Dis 1993; 167:173–9. 8418164 10.1093/infdis/167.1.173

