# Supplementary material for: Empiric Antibiotic Therapy in Suspected Sepsis: Impact of Gentamicin-Based Regimens on Incident Renal Failure and Mortality
Source: Open Forum Infect Dis. 2025 Jun 4;12(6):ofaf319. doi: 10.1093/ofid/ofaf319 (PMC12188214; doi:10.1093/ofid/ofaf319)
Supplement: ofaf319_Supplementary_Data [file ofaf319_supplementary_data.zip › Supplementary_alttext.docx]

Supplementary

**Supplementary Figure 1** | Adjusted probabilities of clinical outcomes according to antibiotic regimens - sensitivity analysis with stages 1-3 combined and stage 4 separated into death without and with AKI

*Alt text:* Bar chart illustrating adjusted probabilities of clinical outcomes according to antibiotic regimens. The chart shows that broad-spectrum β-lactam was associated with a higher level of AKI or death when evaluating stages 1-3 combined and stage 4 separated into death without and with AKI.

**Supplementary Figure 2** | Association between total dose of antibiotic agent and peak creatinine measured within 30 days after admission

*Alt text*: Figure illustrating association between the cumulative dose of antibiotic agent and peak creatinine measured within 30 days after admission, showing no significant association for either broad-spectrum β-lactams (Figure 2A) or gentamicin (Figure 2B).
