# Supplementary material for: Empiric Antibiotic Therapy in Suspected Sepsis: Impact of Gentamicin-Based Regimens on Incident Renal Failure and Mortality
Source: Open Forum Infect Dis. 2025 Jun 4;12(6):ofaf319. doi: 10.1093/ofid/ofaf319 (PMC12188214; doi:10.1093/ofid/ofaf319)
Supplement: ofaf319_Supplementary_Data [file ofaf319_supplementary_data.zip › Supplementary_OFID_040325.pdf]

Supplementary

Supplementary Table 1 | Number of doses and total amount of antibiotics prescribed during admission

Antibiotic agent	Number of doses (median)	Total amount (median)
Gentamicin[1]	2	720 mg
Benzylpenicillin[1]	15	40 g
Ampicillin[1]	8	24 g
Cefotaxime[1]	12	20 g
Piperacillin/tazobactam[1]	10	44 g
Meropenem[1]	17	18 g

1) Antibiotic agents, doses and frequencies recommended by the Norwegian guidelines for community-acquired sepsis during the study period (National guidelines published in 2013):

Gentamicin 5-7 mg/kg once daily (in combination with narrow-spectrum β-lactam) (6-7 mg/kg once daily in current guidelines published in 2022)

Benzylpenicillin 4 g 4 times daily (2.4 g six times daily in current guidelines)

Ampicillin 2 g 4 times daily

Cefotaxime 2 g three times daily

Piperacillin/tazobactam 4/0.5 g four times daily

Meropenem 1 g three times daily

Supplementary Table 2 | Positive blood cultures and the most common bacteria detected according to empirical antibiotic therapy

	Narrow-spectrum β-lactam/gentamicin n = 635	Broad-spectrum β-lactams n = 1282	p-value
Any positive blood culture, n (%)	92 (14.5)	222 (17.3)	0.12
Beta-hemolytic streptococci, n (%)	12 (1.9)	27 (2.1)	0.75
Escherichia coli, n (%)	32 (5.0)	77 (6.0)	0.39
Klebsiella species, n (%)	10 (1.6)	27 (2.1)	0.43
ESBL, n (%)	3 (0.5)	12 (0.9)	0.28
Staphylococcus aureus, n (%)	7 (1.1)	31 (2.4)	0.052
MRSA, n (%)	0 (0.0)	1 (0.1)	0.48
Streptococcus pneumoniae, n (%)	5 (0.8)	7 (0.5)	0.53
Enterococcus species, n (%)	3 (0.5)	19 (1.5)	0.051

Abbreviations: ESBL, Extended-spectrum β-lactamase. MRSA, Methicillin-resistant Staphylococcus aureus.

Supplementary Table 3 | Associations between antibiotic regimen and primary outcome in the sensitivity analysis excluding patients without a discharge diagnosis of infection

	Narrow-spectrum β-lactam/gentamicin (n = 514)	Broad-spectrum β-lactams (n = 944)	Adjusted OR[2] (95% CI)
Primary outcome[1], n (%)			1.58 (1.21 – 2.07)
Stage 0	392 (76.3)	563 (59.6)	
Stage 1	36 (7.0)	76 (8.1)	
Stage 2	4 (0.8)	4 (0.4)	
Stage 3	5 (1.0)	51 (5.4)	
Stage 4	77 (15.0)	250 (26.5)	

1) 5-level ordinal scale. Stage 0 - survival and no creatinine increase. Stage 1 - creatinine increase by 1.5 to 1.9 times baseline or ≥26.5 μmol/L. Stage 2 - creatinine increase by 2.0 to 2.9 times baseline. Stage 3 - creatinine increase by ≥3.0 baseline or ≥353.7 μmol/L or receipt of acute kidney replacement therapy. Stage 4 – death.

2) Ordinal logistic regression. Adjusted for age, sex, CCI score, kidney function (eGFR), time to first dose of antibiotics, and markers of disease severity including National Early Warning Score 2, noradrenaline, respiratory support and admission to intensive care unit or medical intermediate care unit.

Abbreviations: OR, odds ratio. CI, confidence interval.

Supplementary Figure 1 | Adjusted probabilities of clinical outcomes according to antibiotic regimens - sensitivity analysis with stages 1-3 combined and stage 4 separated into death without and with AKI

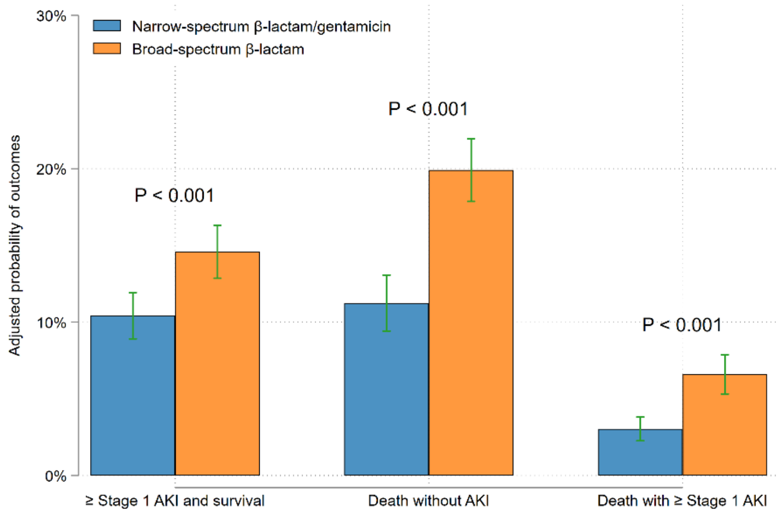

Probabilities dervied from ordinal logistic regression. Adjusted for age, sex, CCI score, kidney function (eGFR), time to first dose of antibiotics, and markers of disease severity including National Early Warning Score 2, noradrenaline, respiratory support and admission to intensive care unit or medical intermediate care unit.

Abbreviations: AKI, acute kidney injury.

Supplementary Figure 2 | Association between total dose of antibiotic agent and peak creatinine measured within 30 days after admission

A) Broad-spectrum β-lactams

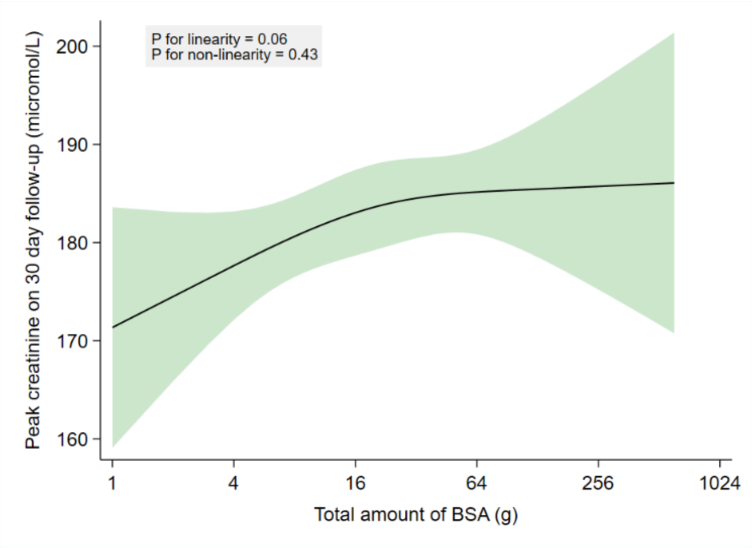

B) Gentamicin

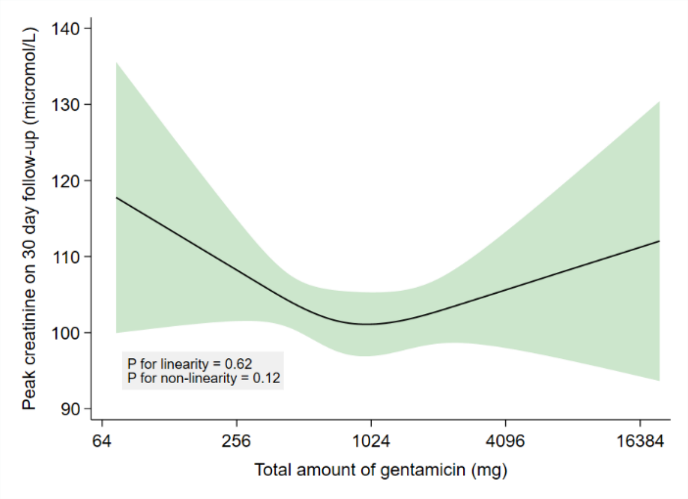

Adjusted for age, sex, CCI, baseline creatinine, renal replacement therapy, time to first dose of antibiotics, and markers of disease severity including National Early Warning Score 2, noradrenaline, respiratory support and admission to intensive care unit or medical intermediate care unit. Dose of antibiotics modeled as restricted cubic splines with knots place at the 10th, 50th, and 90th sample percentiles.
